# Determination of Fluoroquinolones in Pharmaceutical Formulations by Extractive Spectrophotometric Methods Using Ion-Pair Complex Formation with Bromothymol Blue

**DOI:** 10.1155/2018/8436948

**Published:** 2018-10-04

**Authors:** Trung Dung Nguyen, Hoc Bau Le, Thi Oanh Dong, Tien Duc Pham

**Affiliations:** ^1^Faculty of Physics and Chemical Engineering, Le Quy Don Technical University, 236 Hoang Quoc Viet, Hanoi, Vietnam; ^2^Faculty of Chemistry, VNU-University of Science, Vietnam National University Hanoi, 19 Le Thanh Tong, Hoan Kiem, Hanoi, Vietnam

## Abstract

In this paper, we reported a new, simple, accurate, and precise extractive spectrophotometric method for the determination of fluoroquinolones (FQs) including ciprofloxacin (CFX), levofloxacin (LFX), and ofloxacin (OFX) in pharmaceutical formulations. The proposed method is based on the ion-pair formation complexes between FQs and an anionic dye, bromothymol blue (BTB), in acidic medium. The yellow-colored complexes which were extracted into chloroform were measured at the wavelengths of 420, 415, and 418 nm for CFX, LFX, and OFX, respectively. Some effective conditions such as pH, dye concentration, shaking time, and organic solvents were also systematically studied. Very good limit of detection (LOD) of 0.084 *µ*g/mL, 0.101 *µ*g/mL, and 0.105 *µ*g/mL were found for CFX, LFX, and OFX, respectively. The stoichiometry of the complexes formed between FQs and BTB determined by Job's method of continuous variation was 1 : 1. No interference was observed from common excipients occurred in pharmaceutical formulations. The proposed method has been successfully applied to determine the FQs in some pharmaceutical products. A good agreement between extractive spectrophotometric method with high-performance liquid chromatography mass spectrometry (HPLC-MS) for the determination of FQs in some real samples demonstrates that the proposed method is suitable to quantify FQs in pharmaceutical formulations.

## 1. Introduction

Fluoroquinolones (FQs) are the important antibiotics used for the treatment of Gram-negative bacterial infections in both human and veterinary medicine. They are derivatives of 4-quinolone, which have unsubstituted or substituted piperazine ring attached at the 7-position to the central ring system of quinoline as well as fluorine atom at the 6-position. The FQs are useful to treat a variety of infections, including soft-tissue infections, respiratory infections, urinary tract infections, bone-joint infections, typhoid fever, prostatitis, sexually transmitted diseases, acute bronchitis, community-acquired pneumonia, and sinusitis [1–3].

**Figure 1:**
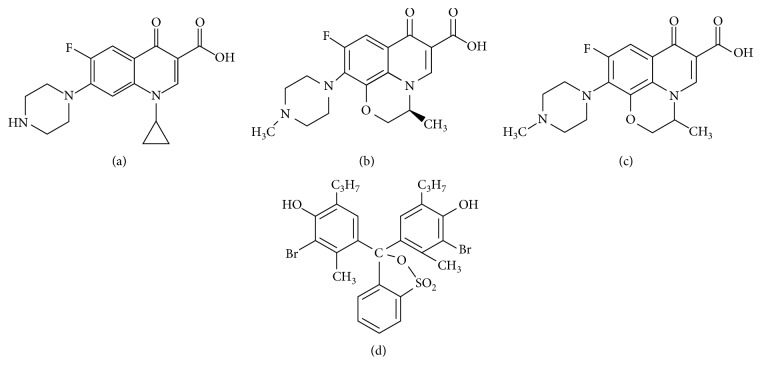
Chemical structures of ciprofloxacin (a), levofloxacin (b), ofloxacin (OFX) (c), and bromothymol blue (d).

Ciprofloxacin (CFX), which is one of the second-generated groups of synthetic FQs, can exhibit greater intrinsic antibacterial activity and make a broader antibacterial spectrum. Ofloxacin (OFX) is a chiral compound that is widely used to treat above infections. Levofloxacin (LFX) is the pure (–)-(S)-enantiomer of the racemic drug substance ofloxacin. Figures 1(a)–1(c) show the chemical structures of CFX, LFX, and OFX, respectively.

Several techniques like voltammetry [4], flow injection electrogenerated chemiluminescence [5], spectrofluorometry [6, 7], spectrophotometry [8, 9], high-performance liquid chromatography [10, 11], and liquid chromatography tandem mass spectrometry [12, 13] have been used for the determination of fluoroquinolones in pharmaceutical and biological products. Among them, spectrophotometric method has several advantages such as simplicity, fast, and low cost. Spectrophotometry was successfully used for pharmaceutical analysis, involving quality control of commercialized product and pharmacodynamic studies. Spectrophotometric methods for the determination of fluoroquinolones could be classified according to the different reactions: (i) charge-transfer complexation based on the reaction of FQs as electron donors with p-acceptors such as 2,3-dichloro-5,6-dicyano-q-benzoquinone, 7,7,8,8-tetracyanoquinodimethane, q-chloranil, q-nitrophenol, and tetracyanoethylene [7, 14–16]; (ii) oxidative coupling reaction using oxidative coupling with 3-methyl-2-benzothiazolinonehydrazone hydrochloride and cerium (IV) ammonium sulfate, Fe(III)-MBTH, tris(*o*-phenanthroline) iron(II), and tris (bipyridyl) iron(II) [17, 18]; (iii) ion-pair complex formation with acid-dye reagents such as Sudan III, methyl orange, supracene violet 3B, tropaeolin 000, bromophenol blue, bromothymol blue, bromocresol green, and bromocresol purple [8, 14, 19, 20]. These methods were related with some major drawbacks such as having narrow linearity range, requiring heating and close pH control, long time for the reaction to complete, and low stability of the colored product formed.

Bromothymol blue (BTB) (Figure 1(d)) is an anionic dye and that can be protonated or deprotonated to form yellow or blue, respectively. The BTB was used to make ion-pair complex, which was applied to determine many pharmaceutical compounds by extractive spectrophotometric methods [21–30]. However, the ion-pair complex between BTB and FQs has not been studied. The method based on ion-pair complexes between analytes and BTB into a suitable organic solvent is also simple, fast, and cheap.

In the previous study, we used sulphonphthalein acid including bromophenol blue, bromocresol green, and bromothymol blue to determine ciprofloxacin pharmaceutical formulations and achieved good results [31].

In this paper, for the first time, we investigated extractive spectrophotometric method based on the formation of ion-pair complexes between ciprofloxacin, levofloxacin, and ofloxacin with BTB subsequent extraction into chloroform. Some effective conditions on the formation of complexes such as pH, shaking time, organic solvent, and the concentration of dye were systematically studied. The present method was also applied to determine FQs in some pharmaceutical formulations including tablets and infusions.

## 2. Experimental

### 2.1. Apparatus

A double beam UV-visible spectrophotometer (SP-60, Biochrom Ltd., UK) with 1.0 cm of path length quartz cells was used to measure all sample absorbances. Inolab pH-meter instrument (Germany) was used to monitor the pH of solutions. Three standard buffers were used to calibrate the electrode before measuring pH of solutions. All measurements were conducted at 25 ± 2°C controlled by air conditional laboratory.

### 2.2. Materials and Reagents

All chemicals used were of analytical grade and double-distilled water was used to prepare all solutions in the present study.

FQs were purchased from Sigma (Germany, with purity >99.0%), whereas bromothymol blue (BTB) was supplied by Maya-R, China, with purity >99%. The organic solvents including chloroform, dichloromethane, carbon tetrachloride, dichloroethane, benzene, toluene, and other chemicals are analytical reagents (Merck, Germany).

The following dosage forms containing FQs were purchased from local pharmacy market and employed in the study: Hasancip and Kacipro tablets equivalent to 500 mg ciprofloxacin (Hasan-Dermapharm and Dong Nam manufacturing-Trading pharmaceutical Co., Ltd, Vietnam). Ciprofloxacin infusion equivalent to 200 mg ciprofloxacin/100 ml solution for infusion (Hebei Tiancheng Pharmaceutical Co., Ltd and Shandong Hualu Pharmaceutical Co., Ltd, China). Stada and DHG tablets equivalent to 500 mg levofloxacin (Stada-VN J.V.Company and DHG pharmaceutical joint–stock company, Vietnam). Ofloxacin (200 mg/tablet) was provided by the Mekophar Chemical Pharmaceutical Company (Vietnam).

### 2.3. Solution Preparation

A stock solution of FQs (1 mg/mL) in double-distilled water. The working standard solution of FQs containing 100 *µ*g/mL was prepared by appropriate dilution. The stock solution of BTB (0.025%) was prepared in double-distilled water. All stock solutions were kept in dark bottle, stored in 4°C and could be used within one week.

### 2.4. Construction of Calibration Curves

A series of 125 mL separating funnel, the volumes of working solutions of the drugs in different concentration ranges (CFX (1–35 *µ*g/mL), LFX (0.5–25 *µ*g/mL), and OFX (0.5–25 *µ*g/mL) were transferred. Then, 4.0 mL of 0.025% BTB solution was added before thoroughly mixing. After that, a 10 mL of chloroform was added to each of the separating funnel. The contents were shaken for 2 min and allowed to separate the two layers. The yellow-colored chloroform layer containing the ion-pair complexes was measured at 420 nm for CFX, 415 nm for LFX, and 418 nm for OFX against the reagent blanks. At each concentration, the experiment was repeated 6 times. The colored chromogen complexes are stable for 24 h.

### 2.5. Sample Preparation

Weigh and mix the contents of twenty tablets of each drug (CFX, LFX, and OFX), an accurately weighed amount of powder equivalent to 0.1 g of drugs transferred into a 100-mL beaker. A magnetic stirrer was used to completely disintegrate the powder in doubly distilled water. Then, filter through a Whatman paper (No 40) and fill up to 100 mL with doubly distilled water in a volumetric flask. The working solution of the drugs containing 100 *µ*g/mL was prepared by dilution and determined under optimum conditions.

### 2.6. Validation with High-Performance Liquid Chromatography-Mass Spectrometry (HPLC-MS)

Some real samples of three FQs were determined by HPLC-MS using HPLC 20 AXL (Shimadzu, Japan) coupled with electrospray ionisation tandem mass spectrometric detection, ABI 5500 QQQ (Applied BioSystem). The chromatographic conditions are including column C18 MRC-ODS (150 mm × 2.1 mm × 3.5 *µ*m), mobile phase containing acetonitrile (ACN) with formic acid (0.1%) in water under a flow rate of 0.5 ml/min, and gradient elution. The inject volume is 10 *µ*L.

## 3. Results and Discussion

### 3.1. Optimum Reaction Conditions

#### 3.1.1. Effect of Extracting Solvent

**Figure 2:**
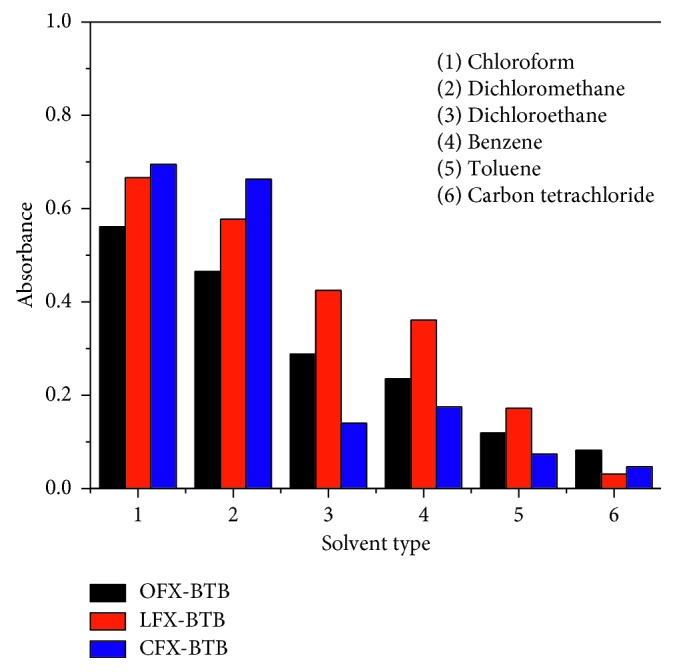
The effect of solvent on the ion-pair complex formation (10 *µ*g/mL of fluoroquinolones (FQs) with bromothymol blue (BTB)).

Six organic solvents including chloroform, carbon tetrachloride, dichloromethane, dichloroethane, benzene, and toluene were used to study the effect of solvent to ion-pair formation between FQs and BTB.  Figure 2 shows that chloroform is the most suitable solvent for the extraction of three FQs with low blank absorbance, highest absorbances, and lowest standard deviations. It implies that chloroform is the best extracting solvent to achieve a good recovery of the complexes with the shortest time to reach the equilibrium processes.

#### 3.1.2. Effect of pH

**Figure 3:**
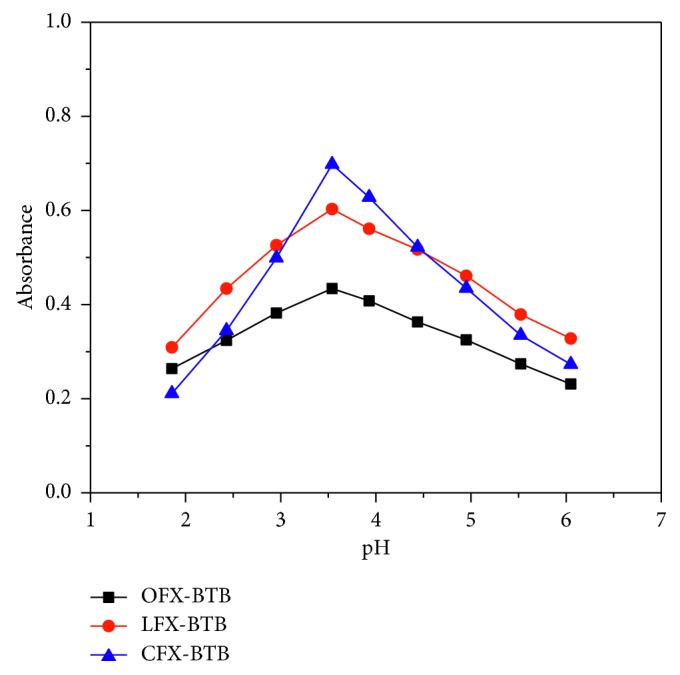
Effect of pH on the absorbances of 10 *μ*g/mL of OFX, LFX, and CFX.

The pH of solution plays an important role in the complex formations. The effect of pH on the formation of ion pairs was examined by varying the pH from 2.0 to 6.0 by adjusting 1 M HCl and 1 M NaOH. The maximum absorbances were observed at pH 3.3, 3.4, and 3.5 for the complexes of BTB and OFX, CFX, and LFX, respectively (Figure 3). These pH values correspond to the initial pH of the examined drug and the dye. Therefore, it is not necessary to adjust the pH before extraction.

#### 3.1.3. Effect of Dye Concentration

**Figure 4:**
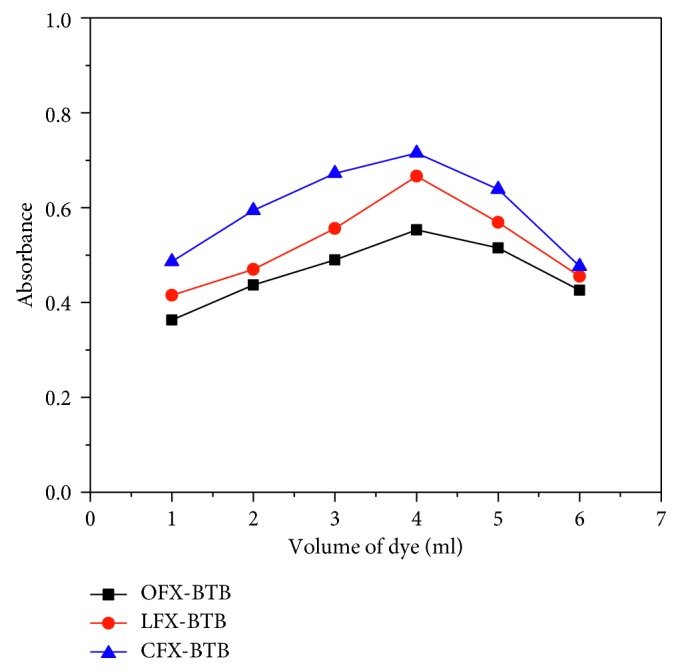
Effect of the volume of 0.025% BTB on the absorbance of 10 *μ*g/mL of OFX, LFX, and CFX.

The effect of dye concentrations was studied by adding different volumes of 0.025% BTB from 1.0 to 6.0 mL with a fixed concentration of FQs (10 *μ*g/mL) (Figure 4). Figure 4 shows that the maximum absorbance of the complex was achieved with 4.0 mL of 0.025% of BTB in each case and excess dye did not affect the absorbance of the complex. Therefore, 4.0 mL of 0.025% of BTB is optimum dye volume and it is kept as constant for further studies.

#### 3.1.4. Effect of Shaking Time

**Figure 5:**
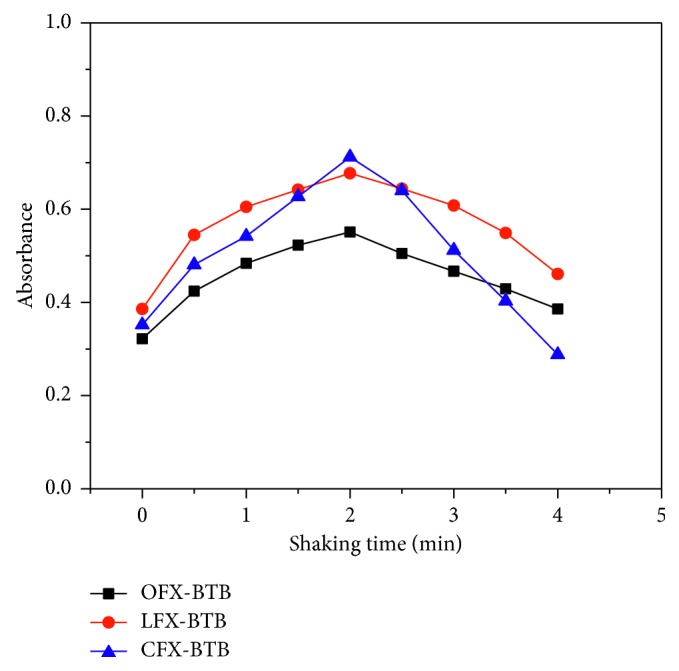
Effect of shaking time on the ion-pair complexes.

The effect of shaking time on the formation and stability of the ion-pair complex was investigated by measuring the absorbance of the extracted ion associates with increasing time from 0 to 4.0 min.  Figure 5 shows that the ion-pair complexes were formed instantaneously with 2.0 min shaking time. Thus, 2.0 min is the optimum shaking time and it is fixed for further studies.

#### 3.1.5. Stoichiometry of Ion-Pair Complexes

**Figure 6:**
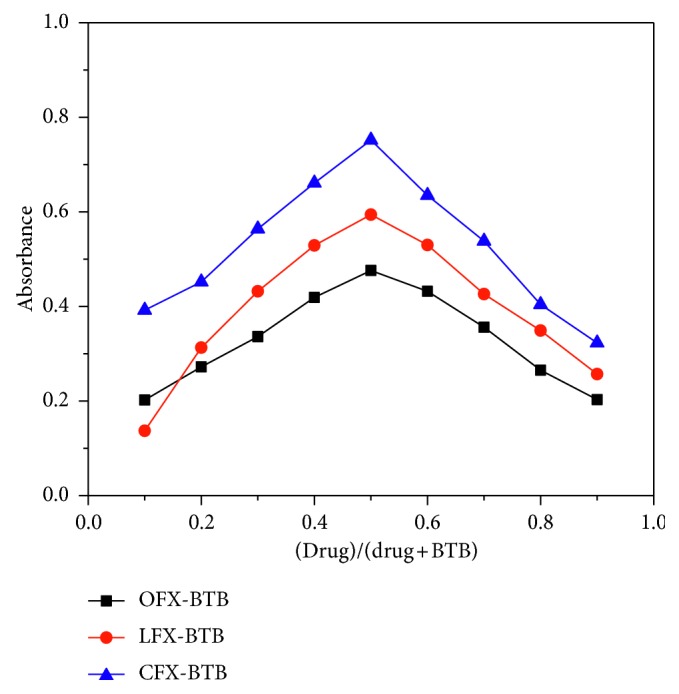
Job's method of continuous variation graph for the reaction of drug with acid dyes BTB, [drug] = [dye] = 3.0 × 10^−4^ M.

Job's method of continuous variation of equimolar solutions was employed to evaluate stoichiometry of the complex. A 3.0 × 10^−4^ M standard solution of three FQs and 3.0 × 10^−4^ M solution of BTB were used. A series of the solutions were prepared in which the total volume of drug and reagent was kept in 10 mL, whereas the absorbances were measured at 420, 415, and 418 nm, for CFX, LFX, and OFX, respectively. The absorbances were plotted against the mole fraction of the drugs. The stoichiometry for each drug-dye ion-pair complex was found to be 1 : 1 (Figure 6).

#### 3.1.6. Mechanism of Reaction and Absorption Spectra

**Figure 7:**
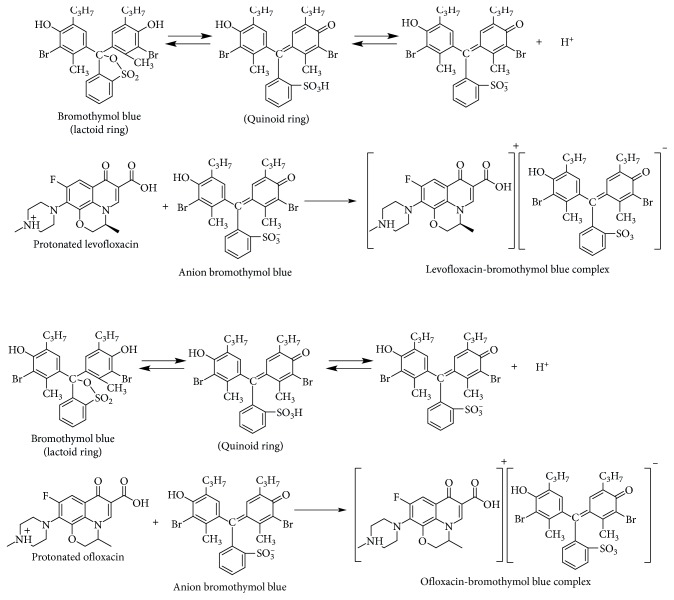
Proposal mechanism for the reaction between levofloxacin, ofloxacin, and bromothymol blue.

Fluoroquinolones can contain a secondary amino group (CFX) and a tertiary amino group (LFX and OFX) that can be easily protonated under acidic conditions. On the one hand, the sulphonic acid group in BTB, that is, the only group undergoing dissociation in the pH range 1–5. The colour of BTB is on the basis of lactoid ring and subsequent formation of quinoid group. It is suggested that the two tautomers are plausible in equilibrium due to strong acidic nature of the sulphonic acid group. Thus, the quinoid body must predominate. Finally, the protonated fluoroquinolones form ion pairs with BTB dye that could be quantitatively extracted into chloroform. The possible reaction mechanisms are proposed and given in a scheme in Figure 7.

**Figure 8:**
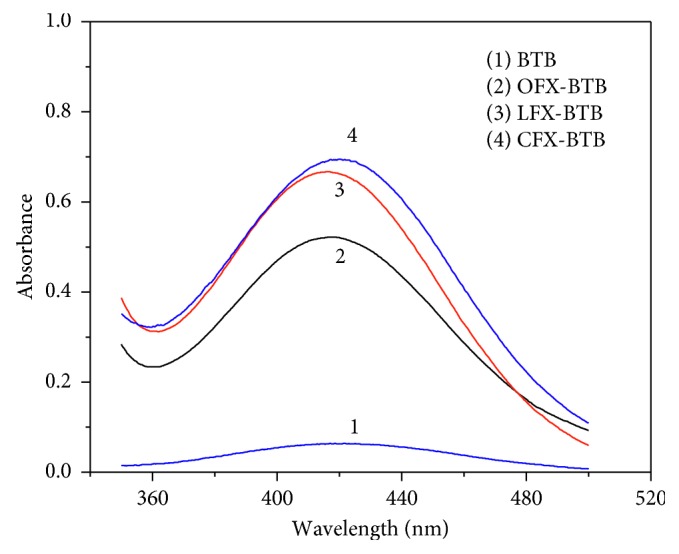
Absorption spectrum of ion-associate complexes of fluoroquinolones (10 *µ*g/mL) with BTB against reagent blank.

The absorption spectra of the ion-pair complexes, which were formed between FQs and BTB, were measured in the wavelength range 350–500 nm against the blank solution and shown in Figure 8.

Figure 8 shows that absorption maxima for CFX-BTB, LFX-BTB, and OFX-BTB in chloroform were observed at 420, 415, and 418 nm, respectively. The reagent blanks under similar conditions have insignificant absorbances. At wavelengths 420, 415, and 418 nm, absorption spectrum of BTB does not affect the absorption spectrum of ion-associate complexes of FQs. Therefore, the selectivity of the proposed method for the determination of FQs is guaranteed.

#### 3.1.7. Association Constants of Ion-Pair Complexes

The equation of association constant of ion-pair complex is

(1) $$\begin{array}{c} \frac{A/A_{\text{m}}}{{\left[1-\left(A/A_{\text{m}}\right)\right]}^{n+2}C_{\text{M}\left(n\right)n}}, \end{array}$$

where *A* and *A*_m_ are the observed absorbance and the maximum absorbance value when all the drug present is associated, respectively. *C*_M_ is the molar concentration of the drugs at the maximum absorbance and n is the stoichiometry in which BTB ion associates with drugs. The conditional stability constants (K_f_) of the ion-pair complexes according to Britton [32] for the cases of FQs were calculated from the continuous variation data using the following equation:

(2) $$\begin{array}{c} K_{f}=\frac{A/A_{\text{m}}}{{\left[1-A/A_{\text{m}}\right]}^{n+2}C_{\text{M}}{\left(n\right)}^{n}}. \end{array}$$

The conditional stability constants (*K*_*f*_) of the ion-pair complexes for FQs are indicated in Table 1.

Table 1 shows that the log K_f_ values of ion-pair associates for OFX-BTB, LFX-BTB, and CFX-BTB were 6.08 ± 0.46, 6.04 ± 0.58, and 5.91 ± 0.32, respectively (numbers of replicated experiments, *n*=6). The obtained results confirmed that the ion-pair formation complexes are of high stability.

### 3.2. Validation of the Present Method

The proposed methods are validated according to ICH recommendations Q2(R1) [33]. The parameters that have been investigated are indicated below.

#### 3.2.1. Linearity, Sensitivity, and Limits of Detection and Quantification

**Figure 9:**
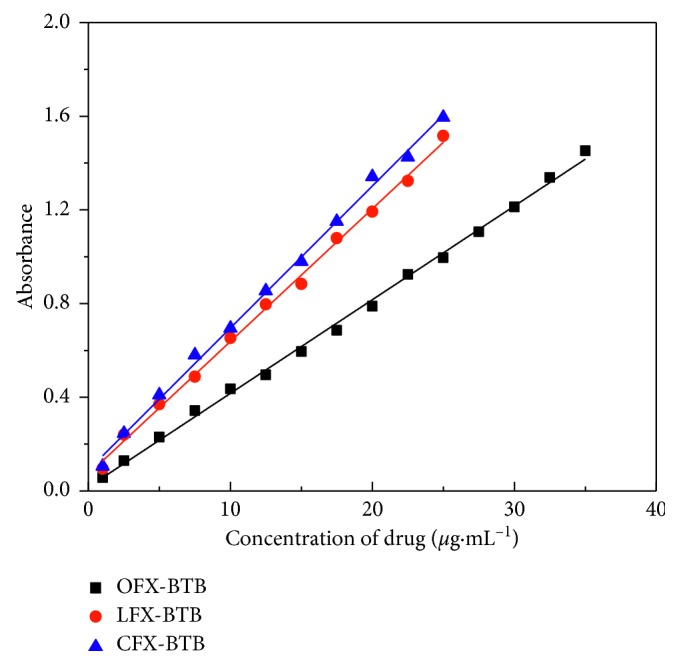
Calibration curves for OFX, LFX, and CFX at 418, 415, and 420 nm, respectively.

A linear relationship between the measured absorbance and the concentration range studied for each drug as shown in Figure 9 and the correlation coefficient (R) of at least 0.997 were achieved. The limit of detection (LOD) and quantification (LOQ) of the method are determined by 3.3(*SD*/*b*) and 10(*SD*/*b*), respectively, where SD is the standard deviation of blank absorbance values and b is the slope of the calibration curve equation.

The LOD and LOQ values, slope, and intercept of linear graphs for all the drugs and analytical parameters are indicated in Table 2. The molar absorptivities and Sandell's sensitivity of each methods were calculated and these values showed that the molar absorptivity of ion-pair complexes was in the order CFX-BTB > LFX-BTB > OFX-BTB.

#### 3.2.2. Accuracy and Precision

The accuracy and precision of the methods were determined by preparing solutions of three different concentrations of drug and analyzing them in six replicates. The precision of the proposed methods was evaluated as percentage relative standard deviation (RSD%) and accuracy as percentage relative error (RE%). The percentage relative error was calculated using the following equation:

(3) $$\begin{array}{c} \mathrm{RE}\left(\%\right)=\left[\frac{\left(\mathrm{founded}–\mathrm{added}\right)}{\mathrm{added}}\right]\times 100. \end{array}$$

The accuracy and precision were summarized in Table 3. The low values of the RSD and RE confirm the high precision and the good accuracy of the present method.

#### 3.2.3. Robustness and Ruggedness

For the evaluation of the method robustness, some parameters were interchanged: pH, dye concentration, wavelength range, and shaking time. The capacity remains unaffected by small deliberate variations. Method ruggedness was expressed as RSD% of the same procedure applied by two analysts and using different instruments on different days. The results showed no statistical differences between different analysts and instruments, suggesting that the developed methods were robust and rugged (Table 4).

#### 3.2.4. Selectivity and Effect of Interferences

The effect of commonly utilized excipients in drug formulation was studied. The investigated FQs were studied with various excipients such as magnesium stearate, glucose, lactose, starch, and sodium chloride which were prepared in the proportion corresponding to their amounts in the real drugs with a final dosage of 10 *µ*g/mL FQ. The effect of excipients on the determination of FQs was evaluated by recovery when determining FQs analyzed with the proposed method in the presence of excipient (Table 5).

The results in Table 5 show that the recoveries are in the range of 98.53–102.04, demonstrating that there is no interference of excipients when FQs in drugs are quantified by extractive spectrophotometric using ion-pair formation with BTB. In other words, the present method has a high selectivity for determining FQs in its dosage forms.

### 3.3. Comparison with Other Spectrophotometric Methods

The proposed method compares with other reported methods. It has been observed that the extractive spectrophotometric method with BTB in the present study is of high sensitivity than other ones (Table 6). It also does not need heating, the product is stable for a longer time, and the interferences are minimum.

### 3.4. Analysis of Pharmaceutical Formulations

The proposed method was applied successfully for the determination of studied drugs in the pharmaceutical formulations (tablets and infusion) and the results are presented in Table 7. Six replicated determinations were measured. Table 7 shows that satisfactory recovery data were obtained and the recovery efficiency varies from 97.41% to 101.20%, indicating high accuracy of the present method in determining real pharmaceutical samples.

### 3.5. Comparison with HPLC-MS Method

In order to validate the experimental data in determining some real drug samples, HPLC-MS was used with the conditions described on Section 2.6 according to the previously published paper [13]. The comparison between the results determined by the present method with HPLC-MS method was indicated in Table 8.

Table 8 shows a good agreement between the proposed method and HPLC-MS where the relative differences of two methods were less than 11%. Furthermore, the standard deviation of the proposed method is almost lower than that of HPLC-MS. Our results indicate that the extractive spectrophotometric determination of FQs using BTB dye in chloroform is a very good method to quantify the FQ in pharmaceutical formulations.

## 4. Conclusions

We have reported a new method when using BTB as an anionic dyes for the extractive spectrophotometric determination of ciprofloxacin (CFX), levofloxacin (LFX), and ofloxacin (OFX) in different pharmaceutical drugs (tablets and infusions). The methods have the advantages of simplicity without heating, pH-adjustment, and high sensitivity. The limit of detection (LOD) values are 0.084 *µ*g/mL for CFX, 0.101 *µ*g/mL for LFX, and 0.105 *µ*g/mL for OFX. No interference from common excipients was confirmed. The stoichiometry complexes of FQs and BTB determined by Job's method of continuous variation were found to be 1 : 1. The developed and validated methods are indicated as the acceptable precision and accuracy, and recovery of the drugs and suitable for routine analysis of drugs in pharmaceutical formulations. The results of some real samples by the present method that were compared with HPLC-MS method with the relative differences are less than 11%, indicating that the present method is good for determination of FQs in pharmaceutical formulations.

## Figures and Tables

**Table 1:** The conditional stability constants (*K*_f_) of the ion-pair complexes for FQs.

Sample	*V*_drug_ (mL)	*V*_BTB_ (mL)	*A*	*n*	*n* ^⋀^ *n*	[1 − (*A*/*A*_*m*_)]^*n*+2^	*K*_f_	log *K*_f_	Mean
Ofloxacin									
1	0.25	2.25	0.202	0.1111	0.7834	0.3116	50204.7802	4.7007	**6.08**
2	0.5	2	0.272	0.2500	0.7071	0.1486	157048.9127	5.1960	
3	0.75	1.75	0.336	0.4286	0.6955	0.0512	572507.7789	5.7578	
4	1	1.5	0.419	0.6667	0.7631	0.0035	9562329.8320	6.9806	
5	1.25	1.25	0.476	1.0000	1.0000	0.0000	_	_	
6	1.5	1	0.432	1.5000	1.8371	0.0002	59414177.6247	7.7739	
7	1.75	0.75	0.356	2.3333	7.2213	0.0026	1172246.1806	6.0690	

Levofloxacin									
1	0.25	2.25	0.137	0.1111	0.7834	0.5749	14789.8587	4.1700	**6.04**
2	0.5	2	0.313	0.2500	0.7071	0.1856	115961.3839	5.0643	
3	0.75	1.75	0.432	0.4286	0.6955	0.0426	708574.1920	5.8504	
4	1	1.5	0.559	0.6667	0.7631	0.0005	67745245.6475	7.8309	
5	1.25	1.25	0.594	1.0000	1.0000	0.0000	_	_	
6	1.5	1	0.53	1.5000	1.8371	0.0004	34165312.8945	7.5336	
7	1.75	0.75	0.426	2.3333	7.2213	0.0042	682897.0665	5.8344	

Ciprofloxacin									
1	0.25	2.25	0.392	0.1111	0.7834	0.2112	83530.0520	4.9218	**5.91**
2	0.5	2	0.452	0.2500	0.7071	0.1265	178145.0100	5.2508	
3	0.75	1.75	0.564	0.4286	0.6955	0.0345	828480.0305	5.9183	
4	1	1.5	0.661	0.6667	0.7631	0.0036	8522213.4055	6.9306	
5	1.25	1.25	0.752	1.0000	1.0000	0.0000	_	_	
6	1.5	1	0.615	1.5000	1.8371	0.0026	4572267.1248	6.6601	
7	1.75	0.75	0.538	2.3333	7.2213	0.0043	608798.6008	5.7845	

—, not determined.

**Table 2:** Analytical characteristics of the proposed methods (*n*=6).

Parameters	Proposed methods		
	Ofloxacin	Levofloxacin	Ciprofloxacin
Colour	Yellow	Yellow	Yellow
Wavelengths *λ*_max_ (nm)	418	415	420
pH	3.3	3.5	3.4
Stability (h)	24	24	24
Shaking time (min)	2	2	2
Stoichiometric ratio	1 : 1	1 : 1	1 : 1
Beer's law range (*µ*g/mL)	1–35	0.5–25	0.5–25
Limit of detection, LOD (*µ*g/mL)	0.105	0.101	0.084
Limit of quantitation, LOQ (*µ*g/mL)	0.315	0.303	0.252
Molar absorptivity (L/mol.cm)	1.44 × 10^4^	2.07 × 10^4^	2.09 × 10^4^
Sandell's sensitivity (*µ*g/cm^2^)	0.068	0.048	0.046

Regression equation (*Y* = *bx* + *a*), where *Y* is the absorbance, *a* is the intercept, *b* is the slope, and *x* is the concentration in *μ*g/mL			
Slope (*b*)	0.040	0.057	0.061
Intercept (*a*)	0.0165	0.072	0.089
Correlation coefficient (*R*)	0.998	0.997	0.998

**Table 3:** Evaluation of accuracy and precision of the proposed methods (*n*=6).

Method	Additive concentration (*μ*g/mL)	Found concentration (*μ*g/mL)	Recovery (%)	RSD (%)	RE (%)
Ofloxacin	5.00	5.11	102.19	2.31	2.2
	10.00	10.26	102.64	1.34	2.6
	15.00	14.89	99.25	0.88	−0.73

Levofloxacin	5.00	5.16	102.70	2.03	2.8
	10.00	10.16	101.56	1.10	1.6
	15.00	14.82	98.80	0.50	−1.2

Ciprofloxacin	5.00	5.13	102.71	1.92	2.6
	10.00	9.74	97.41	0.52	−2.6
	15.00	14.60	97.33	0.57	−2.7

**Table 4:** The results of analysis of pharmaceutical preparation and standard of fluoroquinolones by two different analysts and instruments (*n*=6).

Method	Different instruments			Different analysts		
	X	±SD	RSD (%)	X	±SD	RSD (%)
Ofloxacin-BTB pure ofloxacin (10 *µ*g·mL^−1^) Mekopharm (200 mg ofloxacin per tablet)	10.21	0.19	1.86	9.93	0.24	2.42
	199	0.57	0.29	196	0.76	0.39

Levofloxacin-BTB pure levofloxacin (10 *µ*g·mL^−1^) Stada (500 mg levofloxacin per tablet)	10.16	0.15	1.48	10.19	0.21	2.06
	498	0.61	0.12	501	0.85	0.17

Ciprofloxacin-BTB pure ciprofloxacin (10 *µ*g·mL^−1^) Hasancip (500 mg ciprofloxacin per tablet)	9.85	0.18	1.83	10.12	0.25	2.47
	502	0.64	0.13	497	0.92	0.19

**Table 5:** The effect of excipients on the determination of fluoroquinolones (10 *µ*g/mL).

Recovery (%) ± SD				
Excipients	Amount of excipient added (*μ*g/mL)	Ofloxacin	Levofloxacin	Ciprofloxacin
Magnesium stearate	500	102.04 ± 0.12	101.23 ± 0.089	98.53 ± 0.91
Glucose	250	100.17 ± 0.16	99.04 ± 0.14	99.08 ± 0.062
Lactose	500	99.92 ± 0.21	100.20 ± 0.12	99.73 ± 0.21
Starch	200	100.96 ± 0.24	98.89 ± 0.13	101.31 ± 0.17
Sodium chloride	500	100.13 ± 0.24	100.15 ± 0.11	99.75 ± 0.16

**Table 6:** The comparison of present study with other spectrophotometric methods.

Drug	Reagent	*λ*_max_ (nm)	Range of determination (*μ*g/mL)	Molar absorptivity (L/mol·cm)	Remarks	Reference
Ciprofloxacin	Co (II) tetrathiocyanate	623	20–240	8.38 × 10^2^	Less sensitive	[34]
	Supracene violet 3	575	2.5–30	8.62 × 10^3^	Less sensitive	[35]
	Eosin Y	547	2–8	3.56 × 10^4^	Less stable colour	[36]
	Merbromin	545	2–15	1.23 × 10^4^	Addition of CN^–^to inhibit Hg^+2^ ions	
	Ce(IV)- MBTH	630	10–50	—	Involves shaking time	[17]
	Tris(o-phenanthroline) iron(II)	510	0.04–7.2	3.4 × 10^4^	Involves shaking time and heating	[18]
	Tris (bipyridyl) iron(II)	522	0.05–9	2.95 × 10^4^	Involves shaking time and heating	
	CL	520	16–96	—	Involves shaking time and heating	[16]
	TCNE	335	0.25–15	—	Involves shaking time and heating	
	Sudan II	550	0.8–7.1	5.3 × 10^4^	Narrow linear range	[8]
	Congo red	517	0.5–6.0	2.83 × 10^4^		
	Gentian violet	585	0.5–10	2.21 × 10^4^		
	Brilliant blue G	610	0.5–6.0	2.86 × 10^4^	Narrow linear range and required pH adjustment	[37]
	Bromocresol green	412	1–20	2.28 × 10^4^	Required pH adjustment	[14]
	BTB	420	0.5–25	2.09 × 10^4^	Highly sensitive with wide linear dynamic ranges, no heating, and no pH adjustment	This study

Levofloxacin	Chloranilic acid	521	15–250	1.2 × 10^3^	Less sensitive	[14]
	Bromocresol green	411	1–20	2.16 × 10^4^	Required pH adjustment	
	Eosin Y	547	2–8	4.83 × 10^4^	Less stable colour	[36]
	Merbromin	545	2–15	1.58 × 10^4^	Addition of CN^–^to inhibit Hg^+2^ ions	
	Cobalt (II) tetrathiocyanate	623	20–240	—	Less sensitive	[34]
	Bromophenol blue	424	1.85–31.5	1.98 × 10^4^	Required pH adjustment	[19]
	Bromocresol green	428	1.85–25	1.82 × 10^4^		
	BTB	415	0.5–25	2.07 × 10^4^	Highly sensitive with wide linear dynamic ranges, no heating, and no pH-adjustment	This study

Ofloxacin	Supracene violet 3	575	2.5–25	1.09 × 10^4^	Less sensitive	[35]
	Tropaeolin 000	485	2.5–30	8.23 × 10^2^	Less sensitive	
	Sudan II	560	0.8–8.4	2.97 × 10^4^	Narrow linear range	[8]
	Congo red	530	0.5–5.5	3.29 × 10^4^		
	Gentian violet	575	0.8–11	2.51 × 10^4^		
	Bromocresol purple	400	1.0–16.0	2. 4 × 10^4^	Required pH adjustment	[38]
	Bromocresol green	410	1.0–16.0	1.96 × 10^4^	Required pH adjustment	
	Bromophenol blue	410	5–25	1.03 × 10^4^	Required close pH control and involved extraction steps	[20]
	Bromothymol blue	415	2–15	2.01 × 10^4^		
	Bromocresol purple	410	2–20	1.64 × 10^4^		
	Bromothymol blue	415	1–35	1.44 × 10^4^	Highly sensitive with wide linear dynamic ranges, no heating, and no pH-adjustment	This study

**Table 7:** Determination of the studied drugs in their pharmaceutical preparations using the proposed method (*n*=6).

Pharmaceutical preparation	Hasancip tablet	Kacipro tablet	Shandong infusion	Hebei infusion	Levofloxacin Stada	Levofloxacin DHG	Ofloxacin mekopharm
Labeled amount (mg/form)	500/tablet	500/tablet	200/100 mL	200/100 mL	500/tablet	500/tablet	200/tablet
Recovery (%) ± SD	98.89 ± 0.23	101.20 ± 0.20	97.41 ± 0.42	97.69 ± 0.36	99.53 ± 0.17	101.01 ± 0.35	99.58 ± 0.46

**Table 8:** Amount of some fluoroquinolone antibiotics determined by the proposed method and HPLC-MS.

Sample	Amount (mg/tablet)		Difference (%)
	Proposed method	HPLC -MS	
Ciprofloxacin-Hasancip table	494.45 ± 11.63	446.93 ± 15.84	10.63
Ofloxacin mekopharm	199.16 ± 0.85	202.00 ± 2.72	−1.41
Levofloxacin DHG	505.05 ± 17.33	480.55 ± 54.16	5.10
Levofloxacin Stada	497.65 ± 9.24	486.04 ± 9.24	2.39

## Data Availability

The data used to support the findings of this study are available from the corresponding author upon request.
